# Impulsivity dimensions, emotion dysregulation, and borderline personality disorder features among Italian nonclinical adolescents

**DOI:** 10.1186/2051-6673-1-5

**Published:** 2014-05-12

**Authors:** Andrea Fossati, Kim L Gratz, Cesare Maffei, Serena Borroni

**Affiliations:** Department of Human Studies, LUMSA University, Rome, Italy; Department of Psychiatry and Human Behavior, University of Mississippi Medical Center, Jackson, MS USA; Faculty of Psychology, Vita-Salute San Raffaele University, Milano, Italy; Servizio di Psicologia Clinica e Psicoterapia, San Raffaele Turro, via Stamira d’Ancona, 20, 20127 Milano, Italy

**Keywords:** Emotion Dysregulation, Borderline Personality Disorder, Impulsivity

## Abstract

**Background:**

Contemporary theorists have suggested that impulsivity and emotion dysregulation are two of the core features of BPD. The aim of this study was to evaluate the relationships between Borderline Personality Disorder (BPD) features, impulsivity, and emotion dysregulation in adolescence.

**Methods:**

1,157 nonclinical adolescents were administered the Borderline Personality Inventory, following which three groups of adolescents with high (high–BPD; *n* = 29), average (average-BPD; n = 31), and low (low–BPD; *n* = 31) levels of BPD features were selected. Participants in these three groups were administered the UPPS-P Impulsive Behavior Scale (UPPS-P) and the Difficulties in Emotion Regulation Scale (DERS).

**Results:**

UPPS-P Negative and Positive Urgency scales, as well as the DERS total score, significantly discriminated high-BPD adolescents from both other groups. The differences in UPPS-P Negative and Positive Urgency between high-BPD adolescents and both control groups remained significant when partialing out the variance associated with the DERS; However, when partialing out the variance associated with Positive and Negative Urgency, high-BPD adolescents reported significantly higher DERS scores than only the low-BPD control group (and not the average-BPD group). Finally, although the differences in Positive Urgency between high-BPD adolescents and both control groups remained significant when partialing out the variance associated with Negative Urgency, the between group differences in Negative Urgency did not remain significant when controlling for the variance associated with Positive Urgency.

**Conclusions:**

These findings highlight the relevance of both emotion dysregulation and two dimensions of impulsivity (negative and positive urgency) to BPD features in adolescence, providing evidence for a unique association between BPD features and Positive Urgency in particular. These findings add to the literature in this area, suggesting that the tendency to act rashly in the context of intense positive affect may have unique relevance to BPD features in adolescence.

## Background

Borderline personality disorder (BPD) is characterized by a pervasive pattern of instability in the regulation of emotion, interpersonal relationships, self-image, and impulse control [1, 2], and is frequently diagnosed in both clinical and nonclinical samples [3]. Consistent research data show that BPD is associated with severe functional impairment, high rates of suicide and co-occurring psychiatric disorders, intensive use of treatment, and high costs to society e.g. [3–6]. Notwithstanding the severity of functional impairment associated with a BPD diagnosis, current evidence suggests that the course of BPD may be less stable and distinct over time than was previously believed [7–9].

Studying BPD features in adolescence may be an important means of identifying etiological precursors to BPD and developing more effective prevention and treatment programs [10, 11]. Community-based studies may be particularly useful in improving our knowledge of BPD pathology in adolescence. Indeed, Berkson’s bias [12] suggests that clinic/hospital patients may not only be unrepresentative of the population of BPD cases (e.g., showing more severe PD impairment and perhaps greater Axis II comorbidity), but are also likely to present with greater pathology of all sorts (e.g., Axis I, medical disorders, and other impairments; [13]). Although normal adolescent turmoil may sometimes be misinterpreted as BPD features, there is increasing evidence that BPD can be meaningfully identified among adolescents [14, 15]. The reliability and validity of the BPD diagnosis in adolescent samples have been found to be adequate and largely comparable to those found in adult samples [15]. Further, there appears to be a subgroup of severely affected adolescents for whom the diagnosis of BPD remains stable over time, as well as a less severe subgroup that moves in and out of the diagnosis [14, 15]. One implication of these findings is that BPD pathology in adolescents may be more adequately captured by a dimensional/continuous rather than a categorical approach, as the former may better account for the developmental variability and heterogeneity found among adolescents [15]. Contemporary theorists have suggested that impulsivity and emotion dysregulation are two of the core features of BPD [16]. Empirical research indicates that emotion dysregulation and impulsivity are among the BPD features that are most stable over time [7, 17], and that emerge as the best predictors of self-harm, identity, and interpersonal problems at follow-up [18] and overall BPD psychopathology over time [7, 17].

Notwithstanding the relevance of emotion dysregulation and impulsivity to the understanding and treatment of BPD pathology, researchers lacked an agreed upon and comprehensive definition of these constructs for years. In an effort to advance research in this area, Gratz and colleagues proposed an integrative model of emotion dysregulation [19] that emphasizes the functionality of emotions. According to Gratz’s model [19], emotion regulation may be conceived as a multidimensional construct involving (a) the awareness and understanding of emotions, (b) the acceptance of emotions, (c) the ability to control impulsive behaviour and behave in accordance with desired goals when experiencing negative emotions, and (d) the ability to use emotion regulation strategies flexibly to modulate emotional responses in order to meet individual goals and situational demands [19]. The relative absence of any or all of these abilities is considered indicative of the presence of difficulties in emotion regulation (i.e., emotion dysregulation). It should be noted that according to Gratz and colleagues’ [19] model, the construct of emotion regulation only partially overlaps with the construct of affective instability, which has been shown to significantly predict BPD features in adults [16, 18]. Although the lack of ability to use emotion regulation strategies flexibly to modulate emotional responses [19] may lead to affective instability, rapid emotional shifts may also occur in the context of adequate emotional regulation (provided that responses to these emotional shifts is adaptive and involves the control of behavior in the context of these shifts). Likewise, emotion dysregulation may occur in the absence of intense emotions (in the form of emotional avoidance or a lack of emotional understanding or awareness).

With regard to impulsivity, although Moeller and colleagues [20] proposed a theory-neutral definition of impulsivity as a predisposition toward rapid, unplanned reactions to internal or external stimuli without regard for the negative consequences of these reactions, Whiteside and Lynam [21] recently clarified the multifaceted nature of impulsivity by identifying four distinct components associated with impulsive behavior: Urgency, Premeditation (lack of), Perseverance (lack of), and Sensation Seeking. These dimensions were included in the UPPS Impulsive Behavior Scale [21]. According to their conceptualization, Negative Urgency refers to the tendency to act impulsively when experiencing negative affect, Lack of Premeditation refers to a failure to reflect on the consequences of an act before engaging in that act, Lack of Perseverance refers to an inability to focus or follow through on difficult or boring tasks, and Sensation Seeking refers to an openness to trying new experiences and the tendency to enjoy and pursue activities that are exciting [21]. Additional work with the UPPS has since led to the inclusion of a fifth impulsivity-related dimension: Positive Urgency, which refers to the tendency to act rashly when experiencing an unusually positive mood [22, 23]. Although this trait has shown moderate overlap with Negative Urgency, Positive Urgency has demonstrated unique predictive utility over other facets of the UPPS, predicting higher rates of gambling behavior, illegal drug use, and risky sexual behavior in college students [24, 25]. These findings suggest that the tendency to act rashly under circumstances of positive affect may have an important place in the impulsivity literature.

The relevance of both impulsivity and emotion dysregulation to BPD pathology notwithstanding, contemporary theories of BPD differ with regard to the relative emphasis placed on each. Several theorists have suggested that emotion dysregulation is the primary characteristic underlying other BPD features; from this perspective, impulsive behaviors (e.g., substance abuse, self-harm, etc.) have been conceptualized as attempts to deal with negative emotional states [26–31]. Other theories emphasize the centrality of impulsivity/disinhibition to BPD, suggesting that impulsivity is the core feature underlying the other symptoms of this disorder e.g., [32, 33]. Finally, other theories suggest that it may be the combination of emotional dysregulation and impulsivity that leads to BPD e.g., [11, 34, 35].

Although all of these perspectives have received indirect empirical support (see for a review, [16]), few studies have directly examined the unique contributions of emotion dysregulation and impulsivity to BPD pathology. Chapman, Leung, and Lynch [36] published a seminal paper on impulsivity and emotion dysregulation in BPD, finding that high-BPD participants committed a greater number of impulsive responses and reported greater emotion dysregulation than low-BPD participants. Although the results of this study suggest the relevance of both of these factors to BPD, the authors did not directly test if the association between impulsivity and BPD features was explained by emotion dysregulation, or if it remained significant when accounting for the influence of emotion dysregulation. Likewise, although Tragesser and Robinson [16] found that both impulsivity dimensions of the UPPS (i.e., lack of Premeditation and Negative Urgency) and affective instability additively predicted self-reported BPD features in a sample of undergraduates, they did not examine the role of emotion dysregulation per se. Providing more direct support for the unique roles of both emotion dysregulation and impulsivity in BPD pathology, a recent study examining two large nonclinical samples of Italian adolescents (*N* = 501 and *N* = 1,463) found that both impulsivity and three dimensions of emotion dysregulation (difficulties controlling impulsive behaviors when distressed, limited access to effective emotional regulation strategies, and lack of emotional clarity) were significantly associated with BPD features in both samples [37]. Further, impulsivity scores accounted for a significant amount of additional variance in BPD features above and beyond emotion dysregulation (although the DERS scales explained roughly two to three times more variance in BPD features than trait impulsivity). However, it should be noted that Fossati and colleagues [37] relied on a theory-neutral measure of one dimension of impulsivity (the Barratt Impulsiveness Scale-11-A; [38, 39]), rather than a comprehensive measure of multiple dimensions of impulsivity.

Thus, the goal of the current study was to extend extant research by (a) examining differences in emotion dysregulation and impulsivity (both assessed as multi-faceted constructs) across adolescents with high (high-BPD), average (average-BPD), and low (low-BPD) levels of BPD features, and (b) exploring the unique contributions of emotion dysregulation and impulsivity dimensions to BPD group status within this population. We hypothesized that both the impulsivity and emotion dysregulation measures would significantly discriminate adolescents in the high-BPD group from those in the average- and low-BPD groups. Furthermore, we hypothesized that both emotion dysregulation and impulsivity dimensions would contribute unique variance to BPD group status, such that the observed between-group differences in these factors would remain significant when controlling for the variance associated with the other. The present study did not rely on DSM-IV [40] criteria in order to assess BPD features due to three major considerations: a) researchers have suggested that the DSM-IV [40] emphasis on categorical diagnoses does not adequately represent the latent structure of BPD [41]; b) consistent longitudinal data suggest that the DSM-IV criteria for BPD are not homogeneous, but should be reconceptualized as a hybrid of stable personality traits and intermittently expressed symptomatic behaviors (which could increase the risk of diagnostic inconsistency in adolescents; e.g., [7]; for a review, see [3]); and c) the criteria for BPD were designed for use with adults, and the categorical nature of the criteria make it difficult to conceptualize or identify thresholds for use in adolescence [42]. Thus, we relied on the Borderline Personality Inventory (BPI, [43]) to assess BPD features in our sample.

## Method

### Participants

As part of a broader study on theory of mind and BPD features in adolescents, a total of 1,157 adolescents who were attending public high school were administered the Italian translation of the Borderline Personality Inventory (BPI; [43]) during school time; 572 participants (49.4%) were female and 585 (50.6%) were male, with a mean age of 16.70 years, SD = 1.71 years (range of age: 14–23 years). This sample was completely distinct from past studies examining emotion dysregulation and BPD features in adolescents conducted by this research team (see [37]).

The research protocol of this study received the approval from the LUMSA ethics committee. Ethical principles laid down in the Declaration of Helsinki were followed in the conduct of the study. All participants gave their written consent to participate in the study after it had been explained to them; when participants were of minor age, parents also had to sign written informed consent to allow the participation of their children. All adolescents volunteered to participate in the study. None of them received extra credit or money, the research project was originally presented by principal investigator to the school principle and to the school teacher counsel. Then, the teachers presented the research project to the students as well as to the student’s parents. Finally, the research team was formally presented by the teachers to the students during classroom meetings. The research was presented on personality and its influence on life style The BPI was administered and scored anonymously. In order to subsequently contact participants for administering the measures of emotion dysregulation and impulsivity, each student in the full sample was assigned an alphanumeric code by their teachers; teacher were kept blind to BPI and all other measure scores, whereas the psychologists who administered the measures were kept blind to the students’ identity. Moreover, the psychologists who administered the impulsivity and emotion dysregulation measures were also kept blind to the participants’ BPI scores.

In the full sample, the mean BPI total score was 87.89 (*Mdn* = 85), *SD* = 19.21, with a highest value of 155 (which represents approximately 68% of the BPI maximum score of 204); the Cronbach *α* value of the total score of the Italian translation of the BPI in the full sample was .91. Given that there is no established clinical cut-off score on the BPI for nonclinical adolescents, the high-BPD group in the current study was made up of participants scoring in the upper 3% of the distribution of the BPI total score. The upper 3% cut-off was chosen because it represents the intermediate point of the 1%-6% range of BPD prevalence rates in general population samples e.g., [4, 5]. Based on this criterion, 35 participants with a score of 129 or greater were assigned to the high-BPD group. Likewise, 35 participants scoring 57 or lower (i.e., lower 3% of the BPI total score distribution) were assigned to the low-BPD group. Finally, a sample of 35 participants was randomly selected from the 90 participants who scored between 85 (i.e., the median) and 88 (i.e., the mean); these participants were assigned to the average-BPD group. Of note, six adolescents in the high-BPD group and four adolescents from both the low- and average-BPD groups were absent from school and could not be administered the impulsivity and emotion dysregulation measures. Thus, the final sample sizes were 29 participants, 31 participants, and 31 participants for the high-BPD group, the low-BPD group and the average-BPD groups, respectively.

Although a higher percentage of female participants (*n* = 16, 55.2%) was found in the high-BPD group when compared to both the low-BPD group (*n* = 11, 35.5%), Yates-corrected *χ*^*2*^ (1) = 1.62, *p* > .20, *φ* = .19, and average-BPD group (*n* = 11, 35.5%), Yates-corrected *χ*^*2*^ (1) = 1.62, *p* > .20, *φ* = .19, these differences did not reach statistical significance. Participants’ mean age values were 16.54 years (*SD* = 1.57), 16.81 years (*SD* = 1.70), and 16.68 (*SD* = 1.83) in the high-BPD, average-BPD, and low-BPD groups, respectively; as a whole, these age differences were trivial and did not reach statistical significance, *F* (2, 89) = 1.43, *p* > .20, *η*^*2*^ = .03*.*

### Measures

Participants in the three groups were administered individually the Italian versions of the UPPS-P Impulsive Behavior Scale (UPPS-P; [23]) and the DERS [19] self-report questionnaires. All measures were administered during school time. Participants were administered the UPPS-P and the DERS two to four weeks after the initial BPI [43] administration. All measures were administered in random order. The BPI, the UPPS-P, and the DERS were translated into Italian by two of the authors; for all measures, the adequacy of the Italian translations to their respective original versions was controlled by English mother-tongue professional translators through back-translations.

#### Borderline Personality Inventory (BPI)

The BPI is a 53–item self–report questionnaire based on Kernberg’s concept of borderline personality organization [44]. It assesses a broad range of phenomenological manifestations of BPD pathology, including affectivity and identity disturbances, fear of closeness, interpersonal instability, self–harming/suicidal behavior, impulsive behavior, dissociative symptoms and psychotic symptoms. Applying a modification of the original true–false (yes/no) rating of the items [43], items were scored on a Likert-type scale with four levels of agreement ranging from “disagree strongly” to “agree strongly” [45]. The BPI yields a total score that is a dimensional measure of overall BPD psychopathology (with higher scores corresponding to greater BPD psychopathology). Only the first 51 items of the BPI are summed to compute the BPI total score (which can range from 51 to 204). In adult participants, the BPI has been found to be a reliable and valid measure of BPD, with sensitivity and specificity data showing that the BPI identifies independently diagnosed patients with sufficient hit rates [43]. Likewise, in adult participants, the BPI total score has been shown to yield good agreement with Gunderson’s DIB-R [46, 47] and DSM-III-R [48] diagnostic criteria for BPD [43]. Of particular relevance to this study, the BPI has also been found to be a reliable measure of BPD features in samples of adolescents [45]. In support of the measure’s construct validity, scores on the Italian version of the BPI (Cronbach *α* = .93) have been found to evidence significant associations with self-reports of childhood sexual abuse, *r* = .29, *p* < .001, childhood neglect, *r* = .33, *p* < .001, and emotion dysregulation (as assessed with the DERS), *r* = .39, *p* < .001, in a sample of Italian nonclinical adults (*N* = 431, 58.2% female, mean age = 35 ± 16).

In the present study, the Cronbach α values of the BPI total scores were .91 and .97 in the screening sample (*N* = 1,157) and the three groups combined, respectively. Similar Cronbach *α* values were observed for the BPI total score within each group. In the present study, a subsample of 75 adolescents (41.3% female, mean age = 16.56 years, *SD* = 1.74) agreed to be re-administered the Italian version of the BPI one month after the first administration; the value of the test-retest reliability coefficient was good, *r* = .85, *p* < .001.

#### Difficulties in Emotion Regulation Scale (DERS)

The DERS is 36-item measure that provides a comprehensive assessment of overall emotion dysregulation as well as six specific dimensions: nonacceptance of negative emotions, difficulties engaging in goal-directed behaviors when distressed, difficulties controlling impulsive behaviors when distressed, limited access to effective emotion regulation strategies, lack of emotional awareness, and lack of emotional clarity. The DERS has demonstrated good test–retest reliability and adequate construct and predictive validity [19], and is strongly correlated with an experimental measure of emotion regulation among BPD patients (*r* = -.63; see [49]). Further, the DERS has been found to have good internal consistency and adequate construct and convergent validity among adolescents aged 11–17, as well as a similar factor structure to that found in adults [50]. In the present study, the DERS total score showed adequate internal consistency in the whole sample (*N* = 91), Cronbach *α* = .90, as well as in the high-BPD group (Cronbach *α* = .89), average-BPD group (Cronbach *α* = .78), and low-BPD group (Cronbach *α* = .79).

#### UPPS-P Impulsive Behavior Scale

The UPPS-P Impulsive Behavior Scale [21, 23] is a 59-item self-report measure designed to assess five impulsivity-related traits, including Negative Urgency (12 items), Lack of Premeditation (11 items), Lack of Perseverance (10 items), Sensation Seeking (12 items), and Positive Urgency (14 items). Each UPPS-P item is measured on a four-point Likert-type scale, ranging from 1 (Strongly Agree) to 4 (Strongly Disagree). Whiteside and Lynam [21] found that the UPPS demonstrates excellent internal consistency and convergent validity, and later studies have indicated that the subscales of the UPPS make unique contributions to different disorders (suggesting that these subscales represent important aspects of impulsivity not assessed in other impulsivity measures; [51]). In the present study, Cronbach *α* values in the full sample were .88, .84, .77, .81, and .92 for Negative Urgency, Lack of Premeditation, Lack of Perseverance, Sensation Seeking, and Positive Urgency, respectively. Cronbach *α* values for the UPPS-P scales ranged from .71 (Sensation Seeking) to .86 (Lack of Premeditation) in the high-BPD group, .76 (Negative Urgency) to .86 (Sensation Seeking) in the average-BPD group, and .75 (Lack of Perseverance) to .83 (Lack of Premeditation) in the low-BPD group.

### Data analyses

The assumption of normal distribution of the UPPS-P scale scores and DERS total score was assessed using the Kolmogorov-Smirnov test and Shapiro-Wilks statistic; data were also graphically inspected for outlier detection. The effect of participants’ gender and age on UPPS-P and DERS scale scores was assessed in the context of two-way MANCOVA and ANOVAs, respectively, in which participants’ gender and group membership were entered as fixed factors and participants’ age was entered as a covariate; Pillai *V* was used as a multivariate effect size measure, whereas *η*^*2*^ was used to evaluate the effect size of the univariate *F* tests. In the case of the MANOVA/MANCOVA analyses, the nominal significance level (i.e., *p* < .05) of each univariate *F* test was corrected according to the Bonferroni procedure and set at *p* < .01. The presence of a significant difference between the high-BPD group and each of the control groups (average- and low-BPD) was tested using Bonferroni simultaneous contrasts; in the case of the UPPS-P scales, Bonferroni contrasts were performed only for those scales that showed significant *F* values. If no significant effects of participants’ gender and age were observed, data were re-analyzed using a one-way MANOVA/ANOVA design, and planned comparisons between the high-BPD group and each of the other groups were carried out on raw mean scores. Cohen’s *d*[52] was used as a measure of effect size for Bonferroni contrasts.

In order to examine the unique relations between BPD group status and both DERS and UPPS-P scores, respectively (above and beyond the other), the DERS total score or relevant UPPS-P scores were included as covariates in ANCOVA designs examining between group differences in the other variable, and the significance of planned Bonferroni contrasts was re-assessed using covariate-adjusted means. In analogy with regression-based *P*_*M*_[53] statistic, for previously significant Cohen’s *d* values, we computed a measure of the proportion of mediated effect as the ratio of the difference between the Cohen’s [52]*d* absolute value for a given contrast that was obtained without considering the effect of the covariate and the *d* absolute value for the same contrast that was obtained when including the covariate to the *d* value that was obtained without including the covariate. This measure indicates the proportion of the association between BPD features and both impulsivity and emotion dysregulation, respectively, that can be explained by the other.

## Results

In the whole sample (*N* = 91), Kolmogorov-Smirnov test results suggested that the DERS total score, *z* = 0.90, *p* > .30, and all UPPS-P scale score, min. *z* value (Sensation Seeking) = 0.56, max. *z* value (Lack of Premeditation) = 0.84, all *p*s > .40, were normally distributed; similar conclusions were supported also by the Shapiro-Wilks test results, min. value = .97 (Positive Urgency and Sensation Seeking) max. value (Negative Urgency) = .99, all *p*s > .10.

The UPPS-P scale correlation matrices did not differ significantly across the three groups, homogeneity *χ*^*2*^ (30) = 38.29, *p* > .10; thus, they could be safely pooled. After correcting the nominal significance level according to the Bonferroni procedure (i.e., *p* < .005), significant correlations were observed between Negative Urgency, and Lack of Premeditation, *r* = .36, *p* < .005, Sensation Seeking, *r* = .54, *p* < .001, and Positive Urgency, *r* = .85, *p* < .001; moreover, Lack of Premeditation correlated significantly with Lack of Perseverance, *r* = .74, *p* < .001, and Positive Urgency showed a significant correlation with Sensation Seeking, *r* = .47, *p* < .001.

The DERS total score correlated significantly with all UPPS-P scales even after Bonferroni correction of the significance level (i.e., *p* < .01), with Pearson *r* values of .66, .41, .33, .35, and .60 (all *p*s < .005) for the correlations between the DERS total score and Negative Urgency, Lack of Premeditation, Lack of Perseverance, Sensation Seeking, and Positive Urgency, respectively. No significant differences were observed among the three groups in the *r* values for the correlations between the DERS total score, and Negative Urgency, *χ*^*2*^ (2) = 1.21, *p* > .50, Lack of Premeditation, *χ*^*2*^ (2) = 0.25, *p* > .80, Lack of Perseverance, *χ*^*2*^ (2) = 0.79, *p* > .50, Sensation Seeking, *χ*^*2*^ (2) = 1.79, *p* > .40, and Positive Urgency, *χ*^*2*^ (2) = 0.45, *p* > .50. According to the Box *M* test, the variance-covariance matrices of the UPPS-P scales were homogeneous across the three groups, *M* = 37.13, *F* = 1.09, *p* > .30. According to the hierarchical regression model, the hypothesis of slope parallelism across the three groups for the participants’ age effect on the UPPS-P scales could not be rejected, min. *R*^*2*^_*change*_ = .00 (Lack of Premeditation), max. *R*^*2*^_*change*_ = .02 (Lack of Perseverance), all *p*s > .50. Results of the Two-way MANCOVA did not show any significant effect of participants’ age, Pillai *V* = .08, *p* > .40, or gender, Pillai *V* = .12, *p* > .20, on the five UPPS-P scale scores; the gender-by-group interaction was also not significant, Pillai *V* = .20, *p* > .20. Rather, a significant multivariate group effect on the UPPS-P scale scores was observed, Pillai *V* = .48, *p* < .001. Raw mean scores and *SD*s for the UPPS-P scales in the high-BPD, average-BPD, and low-BPD groups, respectively, as well as univariate *F* tests and *η*^2^ values, are listed in Table 1. The nominal significance level (i.e., p < .05) of each *F* test was corrected according to the Bonferroni procedure and set at *p* < .01; according to univariate *F* tests based on one-way ANOVAs, significant group effects could be observed only for Negative Urgency and Positive Urgency scales; overall group differences accounted for roughly 40% and 50% of the variance in Negative Urgency and Positive Urgency scale scores, respectively.

**Table 1:** **Descriptive statistics, univariate F tests, effect size estimates, and Bonferroni contrasts for the UPPS-P scales and difficulties in emotion regulation scale total score in the high BPD group (n = 29), average BPD group (n = 31), and low BPD group (n = 31)**

	High BPD group		Average BPD group		Low BPD group			
	(***n*** = 29)		(***n*** = 31)		(***n*** = 31)			
UPPS-P scales^1^	***M***	***SD***	***M***	***SD***	***M***	***SD***	***F***	***η*** ^***2***^
Negative Urgency	34.18*	6.50	28.35	5.63	22.57	5.74	19.10***^a^	.38
Lack of Premeditation	24.29	6.28	23.46	5.49	22.83	5.86	0.31^a^	.01
Lack of Perseverance	23.18	4.71	22.50	5.10	21.39	4.94	0.68^a^	.02
Sensation Seeking	33.94	6.98	30.65	7.64	27.09	7.12	4.38^a^	.12
Positive Urgency	39.71*	6.73	30.65	7.49	23.04	6.04	29.22***^a^	.48
DERS Total Score	114.36*	21.53	93.20	14.24	77.30	13.70	35.60***^a^	.46
DERS Total Score (age-adjusted)	113.42	16.50	93.79	16.41	77.58	16.37	34.27***^b^	.45

Note. *DERS* Difficulties in Emotion Regulation Scale, *BPD* Borderline Personality Disorder.

^1^One-way MANOVA Pillai *V* = .50, *p* < .001. ^a^ *F* (2, 88) tests based on one-way ANOVAs. ^b^ *F* (2, 87) test based on one-way ANCOVA.

*High BPD group mean score significantly different (i.e., p < .0083) from both control groups mean scores in Dunn-Bonferroni contrasts. Effect size estimates for Dunn-Bonferroni contrasts: 1. Negative Urgency, High BPD vs. Low BPD: *t* = 9.03, *p* < .001, *d* = 2.33; High BPB vs. Average BPD: *t* = 3.82, *p* < .001, *d* = 0.99. 2. Positive Urgency, High BPD vs. Low BPD: *t* = 9.03, *p* < .001, *d* = 2.33; High BPB vs. Average BPD: *t* = 4.83, *p* < .001, *d* = 1.25. 3. DERS total score, High BPD vs. Low BPD: *t* = 8.42, *p* < .001, *d* = 2.18; High BPB vs. Average BPD: *t* = 4.81, *p* < .001, *d* = 1.24.

***p < .001.

Raw and age-adjusted mean scores and *SD*s for the DERS in the three groups are listed in Table 1. DERS total score variance was homogeneous across the three groups, Levene’s *F* (2, 88) = 2.08, *p* > .10; hierarchical regression models did not reveal any significant group-by-age interaction in the relationship between the DERS total score and participants’ age, thus supporting the hypothesis of slope parallelism across the three groups. The two-way ANCOVA results showed a significant and substantial group effect on the DERS total score, *F* (2, 84) = 34.05, *p* < .001, *η*^2^ = .46, with no significant effect of participants’ gender, *F* (1, 84) = 0.04, *p* > .80, *η*^2^ = .00, or group-by-gender interaction, *F* (2, 84) = 1.97, *p* > .80, *η*^2^ = .05. However, a significant, albeit weak effect of participants’ age on the DERS total score was observed, *F* (1, 84) = 4.64, *p* < .05, *η*^2^ = .06. In the present study, significant group effects were observed for both raw and age-corrected DERS total scores (see Table 1), which explained roughly 45% of the DERS total score variance.

Thus, six Bonferroni contrasts were computed in order to test the significance of the differences in mean scores on Negative Urgency, Positive Urgency, and DERS total scores between the high-BPD group and both the average-BPD and low-BPD groups, respectively. The nominal significance level (i.e., p < .05) of each contrast was corrected according to the Bonferroni procedure and set at *p* < .0083. Scores on both the Negative Urgency and Positive Urgency scales significantly discriminated the high-BPD group from both the low-BPD and average-BPD groups (see Table 1). Interestingly, the between group differences in Positive Urgency remained significant even when controlling for the variance associated with Negative Urgency, *F* (2, 87) = 5.78, p < .005, *η*^2^ = .14, with the high-BPD group evidencing significantly higher Positive Urgency mean scores than both the low-BPD group, Bonferroni *t* = 3.40, *p* < .005, *d* = 0.88, and average-BPD group, Bonferroni *t* = 2.29, *p* < .025, *d* = 0.59. Conversely, the between group differences in Negative Urgency did not remain significant when controlling for the variance associated with Positive Urgency, *F* (2, 87) = 1.50, p > .20, *η*^2^ = .04.

Adolescents in the high-BPD group evidenced significantly higher mean DERS total scores than adolescents in both the low-BPD and average-BPD groups. Similar results were obtained when age-adjusted DERS scores were analyzed, with Bonferroni *t* values of 8.28 (*d* = 2.14) and 4.51 (*d* = 1.17) for the high-BPD versus low-BPD group and high-BPD versus average-BPD group contrasts, respectively.

According to hierarchical regression models, no significant violation of slope parallelism across groups was observed for the relationship between the DERS total score and the UPPS-P Negative Urgency, *R*^*2*^_*change*_ = .00, *p* > .90, and Positive Urgency, *R*^*2*^_*change*_ = .00, *p* > .80, scores. Thus, DERS scores could be safely adjusted using a pooled estimate of the effect of Negative Urgency and Positive Urgency in the ANCOVA model. The mean DERS total scores adjusted for the effects of UPPS-P Negative Urgency and Positive Urgency scales were 103.94 (*SD* = 22.72), 92.95 (*SD* = 16.97), and 83.42 (*SD* = 20.96) for the high-BPD group, average-BPD group, and low-BPD group, respectively. After controlling for the variance associated with Positive and Negative Urgency, the between group differences in DERS total scores remained significant, *F* (2, 86) = 4.84, p < .05, although the *η*^2^ value dropped to .12; according to Bonferroni contrasts, however, the high-BPD group differed significantly from only the low-BPD group on the Urgency-corrected DERS total score, Bonferroni *t* = 3.11, *p* < .005, *d* = 0.80, as the difference between the high- and average-BPD groups did not remain significant, Bonferroni *t* = 2.11, *p* > .0083, *d* = 0.55. The proportions of the effect size for the DERS-BPD relation that can be explained by the variance associated with the UPPS-P Negative and Positive Urgency scales were .63 for the high-BPD versus low-BPD group contrast and .56 for the high-BPD versus average-BPD group contrast.

When controlling for the variance associated the DERS (Pillai *V* = .19, *p* < .001) a significant multivariate group effect was found for Positive and Negative Urgency (Pillai *V* = .29, *p* < .001), with univariate *F* (2, 87) effects of 8.38 (*η*^2^ = .19; *p* < .001) for Negative Urgency and 14.20 (*η*^2^ = .29; *p* < .001) for Positive Urgency. In contrast to the results for the DERS above, all between group differences in Negative and Positive Urgency remained significant when controlling for the variance associated with emotion dysregulation. Specifically, the high BPD group had significantly higher DERS-corrected Negative Urgency scores than both the average BPD group, Bonferroni *t* = 2.70, *p* < .0083, *d* = 0.70 (proportion of effect size that was mediated by the DERS total score = .29), and low BPD group, Bonferroni *t* = 4.09, *p* < .001, *d* = 1.24 (proportion of effect size that was mediated by the DERS total score = .41). Similarly, the high-BPD group had significantly higher DERS-corrected Positive Urgency scores than both the average–BPD group, Bonferroni *t* = 3.41, *p* < .001, *d* = 0.88 (proportion of effect size that was mediated by the DERS total score = .30), and low–BPD group, Bonferroni *t* = 5.33, *p* < .001, *d* = 1.38 (proportion of effect size that was mediated by the DERS total score = .34).

## Discussion

As a whole, our findings confirmed previous findings within adult samples suggesting that emotion dysregulation and some dimensions of impulsivity are robustly related to BPD features in a sample of nonclinical adolescents. Consistent with previous reports e.g., [31, 49, 54–60], emotion dysregulation (as assessed by the DERS total score) significantly discriminated adolescents in the high-BPD group from those in both the average- and low-BPD groups, with effect size values that are considered large by conventional standards [52]. Indeed, even when accounting for the variance associated with Negative and Positive Urgency, DERS scores significantly discriminated adolescents in the high-BPD group from those in the low-BPD group. These findings provide further support for the relevance of emotion dysregulation to BPD and extend the research in this area to adolescents with heightened BPD features.

Our results also extended past research highlighting the relevance of dimensions of impulsivity to BPD [61]. In this study, the tendency to act impulsively when experiencing heightened emotional arousal, both negative and positive, differentiated the high-BPD group of adolescents from both control groups of adolescents. Findings of the relevance of negative urgency to BPD features in this sample of adolescents are consistent with past findings of an positive relation between UPPS Negative Urgency and self-reported BPD features in undergraduates [16], although that study also found a relation between BPD features and Lack of Premeditation on the UPPS. Furthermore, the differences in Negative and Positive Urgency between high-BPD adolescents and both control groups remained significant even when partialing out the variance associated with the DERS. These findings suggest that emotion dysregulation does not fully account for the association between negative and positive urgency and BPD features in adolescents.

Interestingly, despite strong inter-correlations between the Negative and Positive Urgency subscales (consistent with past research, see [62]), these dimensions of impulsivity were differentially associated with BPD features in our sample. Specifically, when controlling for the influence of Positive Urgency, results revealed no significant differences in Negative Urgency between adolescents in the high–BPD group and those in the control groups. Conversely, Positive Urgency significantly discriminated the high-BPD group of adolescents from both control groups even when its overlap with Negative Urgency was taken into account. These findings highlight the relevance of Positive Urgency in particular to BPD features in adolescence, and suggest that Positive Urgency may represent the component of impulsivity that is uniquely altered in adolescents with prominent BPD features. Positive Urgency may be the dimension of impulsivity on the UPPS-P that is most closely related to the construct of “waiting impulsivity,” a form of motivational control that is characterized by the inability to delay rewards and closely related to substance and behavioral addiction [63]. This interpretation is consistent with recent literature suggesting that the decisional and motivational components of impulse control may be more strongly affected in BPD than the cognitive component of impulse control [64].

The particular relevance of positive (vs. negative) emotional arousal to impulse control difficulties among adolescents with heightened BPD features is a novel finding, as most research on BPD focuses on the role of negative emotionality in this disorder. These findings suggest that positive emotional arousal may also increase the likelihood of impulsive behaviors among adolescents with heightened BPD features. As such, these findings have potential clinical implications, highlighting the need for clinicians to assess the frequency and severity of impulsive behaviors that occur during times when adolescents experience a sense of well-being – behaviors that may be less likely to be spontaneously reported as disturbing or problematic by adolescent clients with BPD features (e.g., heavy drinking or sexual promiscuity “just to celebrate” or “just to have fun”).

Interestingly, despite providing evidence for the relevance of Positive and Negative Urgency to BPD features in adolescents, results did not provide support for the relevance of other dimensions of impulsivity to BPD pathology within this population. These findings differ from past research indicating heightened levels of others forms of impulsivity among adults with BPD [65–68], and fail to provide support for theories highlighting the relevance of lack of premeditation to BPD (see [11, 69]). Conversely, these results highlight the importance of emotional arousal to impulsivity within this population, suggesting that the impulsivity most relevant to BPD features in adolescents may be emotion-driven impulsivity that occurs predominantly in the context of emotional arousal (vs. more traditional conceptualizations of impulsivity that focus on the tendency to respond quickly and without forethought in general).

Findings that both emotion dysregulation and Positive and Negative Urgency emerged as uniquely associated with BPD features in adolescents, above and beyond the variance associated with the other, provide support for the role of both emotion dysregulation and emotion-driven impulsivity in BPD pathology (consistent with theories suggesting that it may be the combination of emotional dysregulation and impulsivity that leads to BPD; e.g., [11, 34, 35]). Nonetheless, within our sample, the unique contributions of emotion dysregulation and Positive and Negative Urgency to BPD features were not equivalent. Whereas emotion dysregulation on the DERS accounted for 30-40% of the effect size of the UPPS-P Urgency scales on BPD status within this sample, Negative and Positive Urgency explained roughly 60% of the effect size of the DERS total score on BPD group status. These data suggest that among adolescents with heightened BPD features, the propensity to act impulsively when experiencing either negative (i.e., Negative Urgency) or positive (i.e., Positive Urgency) emotions is not simply a consequence of deficits in other areas of emotion regulation assessed in the DERS (although it is partially related to these). Future research is needed to explore other mechanisms through which emotional arousal influences impulsive behaviors within this population. One possibility is that individuals with BPD pathology may have deficits in the mental representation of emotional states, which in turn may lead to a propensity to act impulsively (see, for instance, [70]).

Our results should be considered in the light of several limitations. The sample was composed of nonclinical adolescents attending high school who volunteered to participate in the study; this strongly limits the generalizability of our findings, particularly with respect to clinical populations of adolescents. In addition, this study relied only on self-reports of BPD features. Although our research design precluded the use of semi-structured interviews for assessing BPD features (due to the need to maintain participants’ anonymity, as well as the requirement of special informed consent when administering psychiatric interviews), concerns about the validity of DSM-IV adult criteria for BPD in adolescents [42] supported the use of a dimensional measure of BPD features in our sample as well. Indeed, concerns have been raised about the current categorical diagnostic system for the classification of personality disorders e.g., [41, 71] and there is increasing support for dimensional models of personality pathology [71–74]. Furthermore, the use of a dimensional measure of BPD features avoids the well-known problems of within-category variation and high diagnostic overlap of a DSM-IV BPD diagnosis [3]. That said, although the BPI does not overlap completely with DSM-IV BPD criteria, it does yield a DSM-based BPD index and has been found to have good agreement with DSM-III-R [48] diagnostic criteria for BPD [43]. Thus, our use of the BPI was considered a more appropriate choice for this sample.

Nonetheless, given that emotion dysregulation and impulsivity were also assessed using self-report questionnaires, the potential influence of shared method variance on our findings needs to be considered. Specifically, it is possible that the use of self-report measures to assess all constructs of interest may have spuriously increased inter-correlations between measures. However, given our interest in examining the unique associations between BPD features and both emotion dysregulation and impulsivity dimensions, shared method variance is less of a concern, and the use of self-report measures for all constructs of interest ensures that method variance did not differentially influence certain findings versus others. Moreover, given the conceptual overlap between Negative Urgency and one dimension of emotion dysregulation as assessed with the DERS (i.e., difficulties controlling behaviors in the context of emotional distress), our results provide a conservative test of the unique relations between impulsivity and emotion dysregulation and BPD features in adolescents. Specifically, findings of unique relations between BPD features and both overall emotion dysregulation and negative urgency (when controlling for the variance associated with the other) suggest that: (a) the relation between emotion dysregulation and BPD features is not fully accounted for by the dimension of emotion dysregulation that overlaps with negative urgency, and (b) the relation between negative urgency and BPD features is robust and only partially explained by the conceptual overlap between negative urgency and one dimension of emotion dysregulation. Nonetheless, future research should include behavioral and/or laboratory measures of emotion dysregulation and these impulsivity dimensions, as research using objective measures of these constructs may result in a different pattern of findings. For instance, Jacob and colleagues [61] found that patients with BPD scored significantly higher than controls on self-report measures of impulsivity, but not behavioral measures of the same constructs. In particular, given the somewhat mixed findings with regard to impulsivity in BPD [61], research examining the precise dimensions of impulsivity (assessed multi-modally) most relevant to BPD pathology in both adolescents and adults is needed. Studies incorporating both self-report and behavioral indices of multiple dimensions of impulsivity and emotion dysregulation would be particularly useful for clarifying the unique relations of these distinct yet inter-related constructs to BPD pathology.

Additionally, the cross-sectional design of our study precludes determination of the precise nature and direction of the relationships examined here. Thus, it remains unknown if emotion dysregulation and positive and negative urgency underlie BPD features in adolescents, or emerge as consequences of these features. Future research should investigate the nature and direction of these relationships through prospective, longitudinal investigations. Finally, given our interest in evaluating the unique contributions of emotion dysregulation and impulsivity dimensions, respectively, to BPD group status in adolescents (above and beyond the variance associated with the other), we chose to focus our analyses on emotion dysregulation and impulsivity per se, rather than co-occurring pathology that may be relevant to one or both of these factors (e.g., major depression or attention deficit hyperactivity disorder [ADHD]). With regard to ADHD in particular, we did not evaluate the co-occurrence between this disorder and BPD features in adolescence for several reasons. First, consistent data (see, for instance, [75, 76]) indicate only a modest overlap between these two disorders in adulthood, with only 16% of BPD patients showing adult ADHD symptoms and only 15% of adults with ADHD receiving a comorbid BPD diagnosis. Higher rates of co-occurrence between these conditions among high school students seem highly unlikely, considering that ADHD severely hampers academic achievement and, in Italy, high school is not compulsory education. Moreover, information on co-occurring ADHD among the adolescents in this sample is not relevant to the primary research question of the unique relationship between impulsivity and BPD features in adolescents, as even findings of high rates of ADHD among the high-BPD adolescents (which is unlikely) could simply reflect an underlying impulsivity contributing to both ADHD and BPD features, rather than speaking to the nature of the relation between impulsivity and BPD features per se. Nonetheless, future research should examine the extent to which co-occurring disorders (e.g., ADHD, depression, avoidant personality disorder, and substance use disorders) moderate the observed associations between BPD features and both impulsivity and emotion dysregulation.

As a whole, these limitations highlight the need for further studies based on a longitudinal design to clarify the precise interrelations of emotion dysregulation, impulsivity, and BPD features in adolescents.

## Conclusions

Despite these limitations, the present findings represent a useful contribution to understanding the role of emotion dysregulation and two related dimensions of impulsivity, Positive and Negative Urgency, in adolescent BPD features.
